# Antenatal care of women who use opioids: a qualitative study of practitioners’ perceptions of strengths and challenges of current service provision in Scotland

**DOI:** 10.1186/s12884-024-06265-w

**Published:** 2024-01-23

**Authors:** T. Hughes, A. McFadden, A. Whittaker, J. P. Boardman, L. Marryat

**Affiliations:** 1 School of Health Sciences, University of Dundee, Dundee, UK; 2 NMAHP Research Unit, Faculty of Health Sciences and Sport, University of Stirling, Stirling, UK; 3 MRC Centre for Reproductive Health, Queen’s Medical Research Institute, University of Edinburgh, Edinburgh, UK

**Keywords:** Opioid-related disorders, Substance use, Pregnancy, Prenatal education, Healthcare disparities, Health inequalities

## Abstract

**Background:**

The increasing rise of women using opioids during pregnancy across the world has warranted concern over the access and quality of antenatal care received by this group. Scotland has particularly high levels of opioid use, and correspondingly, pregnancies involving women who use opioids. The purpose of this study was to investigate the different models of antenatal care for women using opioids during pregnancy in three Scottish Health Board Areas, and to explore multi-disciplinary practitioners’ perceptions of the strengths and challenges of working with women who use opioids through these specialist services.

**Methods:**

Thirteen semi-structured interviews were conducted with health and social care workers who had experience of providing antenatal and postnatal care to women who use drugs across three Scottish Health Board Areas: NHS Greater Glasgow and Clyde, NHS Lothian, and NHS Tayside. Framework Analysis was used to analyse interview data. The five stages of framework analysis were undertaken: familiarisation, identifying the thematic framework, indexing, charting, and mapping and interpretation.

**Results:**

Each area had a specialist antenatal pathway for women who used substances. Pathways varied, with some consisting of specialist midwives, and others comprising a multidisciplinary team (e.g. midwife, mental health nurse, social workers, and an obstetrician). Referral criteria for the specialist service differed between health board areas. These specialised pathways presented several key strengths: continuity of care with one midwife and a strong patient-practitioner relationship; increased number of appointments, support and scans; and highly specialised healthcare professionals with experience of working with substance use. In spite of this, there were a number of limitations to these pathways: a lack of additional psychological support for the mother; some staff not having the skills to engage with the complexity of patients who use substances; and problems with patient engagement.

**Conclusions:**

Across the three areas, there appears to be high-quality multi-disciplinary antenatal services for women who use opioids during pregnancy. However, referral criteria vary and some services appear more comprehensive than others. Further research is needed into the perceptions of women who use opioids on facilitators and barriers to antenatal care, and provision in rural regions of Scotland.

**Supplementary Information:**

The online version contains supplementary material available at 10.1186/s12884-024-06265-w.

## Background

Increasing levels of opioid use in pregnancy across the world have been described as a significant public health concern for both mothers and their children [1]. Evidence indicates that all forms of opioids taken during pregnancy can have impacts on the developing foetus, including adverse birth outcomes, Neonatal Abstinence Syndrome, poorer educational outcomes, behaviour, cognition, hospitalisations, and vision [2–4]. Opioid exposure in pregnancy can be through illicit substance use (e.g. heroin, fentanyl), Medication Assisted Treatment (MAT) (in the UK this primarily comprises prescribed methadone, buprenorphine, or buprenorphine/naloxone )[5], or through opioids given for chronic pain relief (e.g. codeine, oxycodone, etc.). This study focuses on the first two of these groups i.e. women who use illicit substances or MAT in pregnancy.

Antenatal care of women who use substances in the UK is underpinned by guidelines and guidance from the Department of Health and the World Health Organisation [5–7]. Best practice for women who become pregnant whilst using opioids is to encourage and support the women to stabilise on MAT, rather than detoxify completely, due to concerns for both mother and foetus [8, 9]. Additionally, this group of women frequently have other physical and mental health, relationship, and socio-economic complexities in their lives which may impact on their pregnancy [1]. Taken together, these factors mean that pregnancies in this group are usually deemed to be high risk, and input to support women fully throughout pregnancy and in the postnatal period is needed from a wide range of services [10].

The little research that exists on the impact of antenatal provision for this population indicates that lack of access to antenatal care for women who use opioids is associated with poorer outcomes, including a three times higher incidence of perinatal death and low birth weight [11]. However, evidence describes a range of barriers to women accessing care both for their substance use more generally and during the antenatal period specifically. Attitudes towards women who use substances, and the perceived stigma surrounding this, was seen as a key barrier to women accessing services, as demonstrated in previous research [12]. Related to this, women feared having their child removed, particularly if they had already had other children removed from their care, in line with other findings [13, 14]. Logistical issues also featured for women, including lack of suitable childcare, where other children were at home, and transportation to reach appointments [15]. Conversely, women have reported the importance of having practitioners with whom they feel able to build relationships as aiding in their access to services in relation to substance use [13, 15]. To date, however, the majority of empirical research on this topic is based in the US, which has a very different healthcare system, and focusses (albeit importantly) on the views of women accessing services.

Scotland, where this study is set, has high levels of opioid use (notably heroin, methadone and buprenorphine), and the highest recorded rate of opioid-related deaths in the world [16]. Since 1999 in Scotland, a range of area-based services caring specifically for women with high-risk pregnancies and/or women who use substances in pregnancy have been developed. This paper describes different models of antenatal care in Health Boards covering three of the largest urban areas in Scotland and explores multi-disciplinary practitioners perceptions of the strengths and challenges of working with women who use opioids through these specialist services.

## Methods

This paper presents a sub-analysis of a wider qualitative study exploring antenatal and postnatal care pathways, as well as related data recording, for women who use opioids in Scotland (currently unpublished). The current analyses focus only on the exploration of antenatal care pathways for women who use opioids. It aims to describe the models of antenatal care for this group of women and the strengths and challenges of these models from the perspective of practitioners.

### Setting

Antenatal care in Scotland is usually provided by the National Health Service (NHS) and is delivered by the community midwife; for pregnancies deemed to be high risk, this normally in combination with an obstetrician. Women are usually seen around 8-10 times during pregnancy and usually have 2 scans (again high-risk pregnancies receiving more intensive support). Some Health Boards additionally provide specialist antenatal provision for high-risk populations, including women who use substances, as will be described in the analyses below. Three Health Boards were selected because they are among the largest urban areas (and consequently have high proportions of opioid use) in Scotland: NHS Greater Glasgow and Clyde, NHS Lothian, and NHS Tayside. NHS Greater Glasgow and Clyde (GGC) covers a large part of central west Scotland, comprising the City of Glasgow, alongside the surrounding areas of East Dunbartonshire, East Renfrewshire, Inverclyde, Renfrewshire and West Dunbartonshire. NHS Lothian covers the areas of the City of Edinburgh, Midlothian, East and West Lothian, respectively. NHS Tayside provides healthcare for people living in Angus, the City of Dundee and Perth and Kinross.

### Sampling strategy and recruitment

Purposive sampling was initially used to recruit participants from a range of professional backgrounds within each area. Following this snowball sampling was used through asking those interviewed to provide contacts of others in the Health Board that the research team would benefit from talking to. Those contacted were emailed a brief overview of the study and the participant information sheet, to which they were asked to respond if they were interested in participating. Eligibility criteria for participation included having experience of working with women who used opioids during pregnancy, knowledge of either the antenatal or neonatal pathways for women with substance use disorder, or knowledge of the data collection and reporting systems for maternal health and/or substance use at health board and national level. Recruitment took place over 7 months in 2021 and 2022. The research team recruited as many relevant people as they could within each site. At the tail end of the Covid-19 pandemic, this proved challenging, and recruitment was more successful in some health board than others. The participants included: community midwives (*n* = 2), specialist midwives focused on women who use opioids (*n* = 4), health visitors (*n* = 1), neonatologists (*n* = 2), neonatal nurses (n = 1), obstetricians (n = 1), and social workers (n = 2), depending on the practitioners involved in the model of care within each setting. These were spread across the health boards as follows: NHS Greater Glasgow and Clyde (*n* = 4), NHS Lothian (*n* = 2), and NHS Tayside (*n* = 7). Further details of participants are set out in Table 1. Please note that these are not broken down by Health Board or gender, due to potential identification issues.

**Table 1:** Participant characteristics

Role	Field/specialism (w/a)	Experience
Consultant Neonatologist	Care of babies with Neonatal abstinence syndrome	Over 20 years
Consultant Neonatologist	Care of babies with Neonatal abstinence syndrome	Unknown
Consultant Obstetrician	Care of women who use substances	Over 20 years
Health Visitor	Caring for families who use substances	6-10 years
Health Visitor	General	< 1 year in current role Previous experience working within midwifery
Midwife	Maternity ward	< 1 year
Senior Charge Midwife	Specialist midwifery	> 1 year (in current role). Previous experience working within specialist midwifery
Senior Neonatal Charge Nurse	Neonatal Intensive Care Unit	16-20 years experience
Social Work Manager	Adult protection services	Over 20 years
Social Worker	Drug and alcohol services	Unknown
Specialist Midwife	Specialist midwifery	Unknown
Specialist Midwife	Specialist midwifery	Unknown
Specialist Midwife	Specialist midwifery	Unknown

### Data collection

Thirteen individual semi-structured interviews were carried out between November 2021 and August 2022, and lasted approximately 20 to 45 minutes (mean 25 minutes). The first author conducted all interviews and was trained in social science methods including semi-structured interviewing. Interview questions and prompts on the interview guide (Additional file 1) were based on a review of the literature and discussions with experts in the field. During the interviews, participants were asked about their experience of working with women who use opioids during pregnancy, the level and nature of substance use in their area, the current antenatal pathways available for this population, the impact of Covid-19 on the antenatal pathways, perceived strengths and gaps in the antenatal pathway, current data reporting methods and storage systems, gaps in the data reporting and storage, and the management of Neonatal Abstinence Syndrome (NAS) in their Health Board. Due to Covid-19 and the related strains on the NHS, all interviews took place via Microsoft Teams or by telephone call. All interviews were recorded and transcribed.

### Data analysis

Framework analysis [17] was used within NVIVO v12 to analyse interview data. The five stages of framework analysis were undertaken [17]: 1) familiarisation with data collected in interviews through immersion in the data by listening to recordings and reading transcripts; 2) identifying the thematic framework through a priori issues (i.e. those informed by the research aims and introduced into the interviews via the topic guide), alongside emergent issues raised by the respondents which became apparent as the framework was initially tested on a few transcripts; 3) indexing, where by the framework is systematically applied to the transcripts; 4) charting, where by relevant aspects of the text are summarised into the framework chart for each case; and 5) mapping and interpretation, in which the researcher explored data held within the framework chart to understand and compare and contrast the nature of substance use in each area, different models of antenatal care in each health board area, and the perceptions from different points of view within and across areas, as well as investigating the impact of Covid-19 on antenatal care pathways, and the perceived strengths and challenges of these pathways. TH and LM both carried out stages 1,2, 3, and 5, whilst LM carried out stage 4.

## Results

Two key topics were described within the framework: models of antenatal care (including the impact of the pandemic), strengths and challenges with current provision. These will now be explored in turn.

### Models of antenatal care provision by health board

Within the three Health Boards investigated, each contained a specialist service which catered specifically for women who use opioids. These services varied, however, each comprising a different mix of health professionals and operating in different ways, for example in their referral criteria and service provision antenatally and, in some cases, postnatally. It should be noted that although participants were asked specifically about women who use opioids in pregnancy, many services covered substance use more broadly, and participants often spoke in these broader terms. A profile of the different service sites and models of care, as described by participants, is presented below.

#### NHS Greater Glasgow and Clyde

In NHS GGC women who use substances are assigned to the Special Needs in Pregnancy Service (SNIPs). The service was initially established for women with ‘addiction problems’ in the 1990s, however, the remit of the service expanded to include women with mental health issues, as well as other vulnerabilities such as homelessness or gender-based violence, with the majority of women now being referred for mental health difficulties. The SNIPs team comprises specialist midwives and an obstetrician. Women are typically referred by either Alcohol and Drug services or community midwives. Other professionals, such as the Police and Accident and Emergency (A&E) teams are also reported to refer women to the SNIPs service.

The service operates out of three hospitals in the GGC area: The Princess Royal Maternity Hospital, the Queen Elizabeth University Hospital, and the Inverclyde Royal Hospital. Women are assigned to a SNIPs team member based on their postcode and have a designated midwife who will see them throughout their pregnancy as well as postnatally. In instances where their midwife is unavailable, the clients will meet with another member of the SNIPs team.

*“Each of the team members of SNIPS are allocated certain postcodes, so that referral will go to the midwife who’s allocated to that postcode, to basically problem-solve and gain some further information. Then we would offer them an appointment with the SNIPS team. The idea is that they get a continuity of care so once they’re in with the team, they tend to see that named person or another member of the SNIPS team in their absence, and that’s just to kind of obviously make sure that everything that needs to be done is being done and we have a regular review of seeing those women.*” (Midwife- Greater Glasgow and Clyde)

All healthcare workers identified that the women have more frequent visits, scans and tests with the SNIPs team throughout their pregnancy, than they would in usual antenatal care. Women were reported to be seen at 10 or more appointments, compared with 8 appointments for women with low-risk pregnancies. Additionally, women were offered extra appointments based on individual need and were able to call their midwife at any time, and the team will conduct home visits to women as well, which was described as helpful for midwives to see the environment that people are living in. Women were free to leave the service at any point and return to the usual antenatal care pathway.

The participants described a close working arrangement between the SNIPs team, drug and alcohol workers, and social workers. Communication between the three services was reported to be very good. SNIP midwives may refer clients to drug and alcohol services and social work services, and there were regular liaison meetings between the three services. The frequency of these meetings differed based on the local area, however, typically they met monthly or once every three weeks. In Renfrewshire, all pregnant clients with substance use issues are allocated a single specialist addictions nurse, who liaises with the pre-birth social work team.

*“In Renfrewshire, we’re very lucky that all our girls that are open to addictions have their care transferred over to a specialist addictions nurse that works with the pre-births social work team. So, they get specialist input from somebody that’s used to dealing with pregnant women. Most other authorities just continue with their routine, addiction support but here in Renfrewshire it’s a bit more specialist.”* (Midwife- Greater Glasgow and Clyde)

Covid-19 was reported to have a relatively minor impact on the delivery of the SNIPs service. There was a slight reduction in the number of home visits, however midwives were still able to visit for child protection concerns and to check on the wellbeing of the mother. Whilst psychiatric appointments moved to being entirely virtual, obstetric appointments for SNIPs women remained in-person, due to women reporting that they found it difficult to get their point across when meetings were not face to face. Communications between the SNIPs team and Alcohol and Drug Recovery Service (ADRS) were slightly reduced and converted to remote working. There was a view by midwives that the interdisciplinary meetings held virtually were one of the biggest challenges facing parents during the pandemic:

*“A lot of the other authorities, the meetings are by telephone, and obviously it’s a very stressful difficult situation for patients to be in any time but for it to be at the end of a telephone, I think it’s pretty bad. And they don’t know who’s speaking, and sometimes they’ve not met the people who are at the meeting because they’re maybe, for example, health visitors or people that are standing in for the workers that they’re used to speaking to or seeing.”* Midwife- Greater Glasgow and Clyde)

Referrals to the SNIPs team were affected by the pandemic, due to the SNIPs team’s reliance on community midwives to gather information about substance use and other potential issues during the booking appointment. When booking appointments moved to being conducted by telephone a decision was made not to ask questions about social circumstances, as they did not know whether the woman was safe to answer in her setting (e.g. was a partner or other person present), and although these questions should have been asked at a later stage, there was a concern that some women may have been missed:

*“So, the social questions were then supposed to be asked when patients came into hospital, but quite often that wasn’t done. The idea was at their first hospital visit that would be gone over again, but of course by the time they got to hospital nobody remembered that that wasn’t done. So, for two years probably quite a lot was missed. But they are now reverting back to face-to-face bookings, so hopefully that will no longer be an issue.”* (Obstetrician – Greater Glasgow and Clyde)

Whilst a further issue, noted to have been since resolved, was the higher levels of aggression from visitors accompanying pregnant women, which at one point was described as being ‘really quite bad’.

#### NHS Lothian

Within NHS Lothian a specialist service for pregnant women who use substances, based in Edinburgh city, was created in 2004, called the PrePare team. This is led by a senior social worker as part of the City of Edinburgh Council social work department (in contrast to the other programmes to be discussed) and was described as a multidisciplinary team spanning pregnancy and the early years and incorporating midwifery, health visiting, community mental health nursing, addiction services, and Early Years officers, the latter of whom support pregnant women and their partners in becoming parents, deliver the ‘Parents Under Pressure’ programme (a specialist parenting programme for parents who use substances) [18] and undertake parenting assessments for children and families social work teams. Of note in this model is that the team work with fathers who use substances as well as mothers.

*“the idea is it’s kind of like a one-stop shop, in the sense that they come into the team, and they’ll get support with their maternity care, with their mental health, with their addiction and like with their parenting support”* (Midwife - Lothian)

Women can be referred by any other service, with health professionals reporting that referrals tend to come from social work, GPs, addiction services, other midwives (i.e. outside the PrePare team), and third sector organisations. Referral criteria were reported as being aged over 16, pregnant and with ‘chaotic substance use’. The service is available to parents living in the City of Edinburgh, but not the other Local Authorities in Edinburgh (Midlothian, East Lothian or West Lothian). Although referral criteria haven’t changed, a change in the demographics of women coming into the service has been noted, with increases in women with crack cocaine usage, alongside histories of heroin use, being seen, and a rise in alcohol use associated with women in their twenties/early thirties.

Women within the service were reported to receive more ‘flexible’ care than usual midwifery provision, and to received additional scans and consultant obstetric appointments. Whilst some women receive all their drug use and antenatal care through the PrePare service (including prescriptions for Medication Assisted Treatment), some women on Drug Treatment and Testing Orders would be required to attend drug services for their prescription but would receive antenatal care through PrePare still. Midwives see women in their own home or another venue in which they feel comfortable, for example their GP practice or addiction service location. It was noted that this was particularly challenging at the start of the pandemic when many of their usual venues were closed and it was unclear whether home visits were allowed:

*“when everything happened with Covid and the GP surgeries shut down and we weren’t really sure what we were to do with regards to home visits and things it was very, very difficult with how we were going to actually see our women. A lot of our women are homeless as well, so it wasn’t necessarily possible to go back into the B and Bs and into hostels and things during that time. And with our women being homeless we don’t necessarily have one GP surgery that we could use, so we were trying to work out which GP surgery would let us in and which GP surgery would let us bring other women from different surgeries in so we could actually provide care.”* (Midwife, Lothian)

#### NHS Tayside

Antenatal care for women who use substances in Tayside varies within the Health Board. Dundee is the only part of the Health Board which has a specialist service for women who use substances; the New Beginnings service. The service was set up in 2010 for women with ‘problematic substance use’, but now has been extended to other vulnerable women, for example those with learning disabilities. The service consists of a multi-agency team comprising five social workers (children and families service), a community mental health nurse, a community midwife, an employment worker, a learning disabilities nurse, a drug worker, and an obstetrician. Referral to the New Beginnings team is typically made by the woman’s social worker or Drug and Alcohol recovery service. The criteria for admission to this pathway is that the woman has no other children at home (i.e. this pregnancy is her first child *or* all other children have been removed) and that she lives within Dundee City. Women with substance use who live outside of Dundee or do not meet the referral criteria have no access to a specialist antenatal service but instead receive antenatal care through the community midwife as usual.

As in the previous areas, the service was described as intending to be a ‘one-stop shop’:

*“So basically… if [women] need to see social work they can come in or we can go to their house, but they can see the social worker and if they need any help with their mental health we’ve got [Community Mental Health Nurse] available to help support them with that, when they’re pregnant I’m available to help them support their pregnancy and early postnatally as well. We have obviously, when they’re on with drug services, what happens is their care is normally transferred from their existing drug worker to our drug worker, so that the whole thing is with us when they’re pregnant to make it easier”* – (Midwife- Tayside).

Working alongside the specialist pathway, multi-agency approaches were used to provide assistance for women beyond the realms of antenatal care. Social workers and midwives frequently worked together, for example when midwives required toxicology screenings, social workers were reported to mandate such screenings:

*“the protocol is that you do toxicology screening and we can't force people to do it. And quite often they don't consent to that. And they say like, no, you're not gonna. I don't want you to do that…so we can't force women, but social work can. And they can make them and they can enforce that.”* (Midwife, Tayside).

During instances of domestic violence, midwives further worked with social workers to provide referrals to third sector organisations for trauma support and organisations such as Women’s Aid. Throughout this multiagency team working, social workers were able to share information and case notes with the New Beginnings service facilitated by the city council IT systems. The New Beginnings team also had strong relationships with Health Visiting teams: for example, when a child was to be fostered, health visitors would support the foster parents/carer and have increased liaison with the neonatal ward. Collaborations between health visiting and third sector organisations and ‘Mum and Me’ (parent peer support) groups were also identified in order to help mothers with community integration.

Covid-19 had some impact on New Beginnings service, although the majority of the service was reported to have remained in person. Participants did describe difficulty accessing women via telephone to arrange appointments, however, and decreased time and resources to visit the women, sometimes making it challenging to contact women. Indeed, resources were said to be ‘completely stretched’ because of the pandemic, primarily due to staff sickness. As in other settings, multi-agency meetings moved to being held online, although there was a perception in Tayside that this was beneficial for some families:

*“…our client group potentially [were a] wee bit more at ease not being stuck in the same room as folk and you know are… less intimidated by 15 professional sitting around the room. Teams are a bit less intimidating. So those things have actually worked quite well as opposed to being a negative, although it was a negative at first because the infrastructure wasn't in place, but now things are running pretty well.” (Social worker – Tayside)*

Additionally, there were some knock-on effects of changes to third sector provision, with the New Beginnings team retaining women within the service beyond the usual period due to lack of third sector support to refer women onto.

### Strengths and challenges of the current approaches

Several strengths of the current antenatal care provision for women who use substances were identified by the health professionals involved in the study. For women included in the specialist services, one of the key strengths reported was continuity of care for women. Women were assigned to a specialist midwife, who they would typically see during and after their pregnancy. Having one assigned midwife was designed to develop a trusting relationship between the woman and midwife to promote open communication, thereby preventing any sudden crises, or non-disclosure of important information. In some local areas, women on specialist pathways were additionally assigned specialist addiction and/or social workers. This was said to be beneficial compared with standard services where women would typically see many different practitioners, which required women to re-tell their story at each appointment:

*“…so it’s just really about building up relationships and … we tend to find that, because they’re not having to repeat the same story 40 million times … to different people, and we don’t have to refer them out, so if the social worker thinks, “I’m not too happy about their mental health” then our mental health worker can have a chat with them … and [they] can provide support without us having to refer them to the community mental health teams, we can provide that in-house. Somebody’s got a learning disability again, we can do that in-house because they can be assessed by [Learning disability nurse], and [they] can offer … support that’s needed for that as well … And then obviously we’ve got our own drug worker as well, so … we, they keep them all the way through their pregnancy and again right up until … the babies are one, and then they’re transferred back to services, and … the feedback from the women seems to be they feel it’s the first time they’ve been listened to, and they know who they’re going to get and it’s the same person all the time, and … they’re not having to try to deal with lots of different people, they know they’ve got stability”* (Social worker, Tayside)

Care within the specialist services was both more individualised and more intense according to participants in the study. Women were reported as being given more personalised care based on their situation and their and their baby’s needs. Health professionals within the specialist services perceived that women engaged well with antenatal services in general because the women cared about outcomes for their baby. Where the services were struggling to reach women, substantial efforts were made to recontact women: midwives reported going to town centres or pharmacies where women are known to attend, or attending women’s scans, which were the most frequently attended appointments, even when other antenatal appointments were missed.

*“…it's happened more than once where [the midwife’s] driven into the centre of town to look for people and she's really in the know about… how these people live… And she makes a point of trying to reach these people.”* – (Midwife, Tayside)

*“…what we have to do is constantly phone them, send them texts the day before, the day of their appointments do you know, to make them attend, attend their appointments and I often, once they’re booked and they’re on the system, as a rule of thumb they generally attend for their scans because they like to see what’s going on, but sometimes getting them to engage after that can be quite difficult, and … I spend half my life, to be honest with you, trailing all over Dundee looking for folk… or … hanging about outside chemists trying to get a hold of women to get them to engage”* (Social worker, Tayside)

Home visits were also conducted by the team to help get a better understanding of the home environment or to make women more comfortable. Care typically included additional midwife visits and scans, compared with the standard antenatal services. Women could request further support before scheduled appointments or call their midwife if they had any queries or concerns. Additionally, the specialist midwife in Tayside provides further training for community midwives outwith specialist teams to upskill them in working with women who use substances in the community.

Communication between the specialist teams and partner services (i.e. NHS Substance Use services and social work) was an additional strength of the pathways. One view was that interdisciplinary working between health visitors and substance use services had improved in recent years due to improved e-health systems. By contrast, another view from a different area was that communication between these teams remained problematic, and that it could be difficult to get information from the addiction team about the woman’s prescription, engagement with addiction services and toxicology results. In addition, some Health Boards used different IT systems in the neonatal ward, and thus reported challenges in information sharing at that stage.

Services also faced challenges in the delivery of antenatal care to women who use drugs. One of the main challenges identified was ‘poor engagement’. Women were perceived as living “chaotic” lives, with midwives noting difficulty accessing them via telephone call or digitally. There were perceived limits in the type of support able to be offered to women and barriers to women accessing the support which was available. There was a perceived lack of support for dealing with trauma, psychological support more widely, and practical support for women. There was a feeling that social work support tended to prioritise the child. For some women, there was an acknowledgement that there might be resistance to further social work involvement due to historic experiences with social work, for example, around child removal. An additional suggestion was the introduction of residential programmes during pregnancy, as seen in other countries, such as Sweden. These services aim to reduce the risk of harms associated with maternal opioid use and offer practical and emotional support. One view was that a lack of funding was the biggest constraint in accessing additional external resources, e.g. residential rehabilitation. Outwith the specialist services, it was felt that community midwives lacked skills in engaging with this specific clientele and their needs, and there was a need for further training in how to deal with the complexities and vulnerabilities of the caseload.

Women were further perceived as facing barriers to accessing perinatal mental health services, particularly where mental health support was not integrated in the specialist pathway: women were reported to be referred often to addiction psychiatry services instead of perinatal mental health services, and these services were not set up to see patients in the timescale of a pregnancy. This was thought to leave women without specialised mental health support and created additional barriers to tackling their substance use. Several healthcare professionals reported problems with linking women to the necessary support and service once additional support needs had been identified:

*“The one difficulty is mental health support for these women… We have available to us the perinatal mental health team, and the specialist psychiatrist for Glasgow, but addiction is something which they do not see, so they would prefer to point our mothers towards addiction psychiatry. But addiction psychiatry are not geared up to see patients in the timescale of a pregnancy, so these women very much fall into a hole.” (Obstetrician- Greater Glasgow and Clyde)*

A commonly identified challenge was a concern that women were reluctant to disclose their substance use, or the full extent of their substance use, with healthcare staff. This was thought to make it challenging for healthcare workers to tailor care to the patients.

## Discussion

This paper described the models of antenatal care for women who use opioids in pregnancy, focusing on three Health Boards in large urban areas of Scotland. Each area was described as having a specialist service for women who used substances, including opioids, and these had tended to broaden over time to include women experiencing other types of adversity or challenges in pregnancy. Although there were overlaps in the operation of these services, for example all having a more intensive antenatal care package being provided by specially trained midwives, other elements varied between Health Boards, such as the interdisciplinary nature of the teams and the leadership by different groups (e.g. health and social work) within that, and the referral criteria, for example in Edinburgh, not all women who use drugs are seen - only those who meet the ‘higher risk’ threshold. Antenatal health care is seen as being unequivocally beneficial for maternal and child outcomes during pregnancy, birth, and the postnatal period [19, 20]. This is even more the case in high-risk pregnancies, such as those for women who use opioids [11]. Several models of care for pregnant women with substance use have been demonstrated to produce positive impacts on outcomes such as birthweight, preterm birth, placental abruption, and prevalence of Neonatal Abstinence Syndrome [21]. It should be noted however that the outcomes for all models of care analysed in Johnson’s [21] systematic review were based in the US or Canada, where care as usual is very different to the UK where all women are entitled to antenatal care, addiction and child health services, even if they do not have access to a specialist service. As with the case study areas explored in our study, the successful models of care in Johnson [21] had a focus on multi-agency working, particularly between addiction services and midwifery/obstetrics, and this is highlighted as best practice within the most recent UK clinical guidelines [5]. It is notable though that the evidence around the specific impact of integrated models of care versus specialist services (e.g. specialist midwifery alone) for women who use substances more generally is largely unknown: women appear to find specialist services more acceptable, and thus attendance and retention rates are often better, which is likely in itself to result in better outcomes [21].

Several strengths of the models of care within the case study areas were perceived by health practitioners working with women who use opioids: these included the continuity for women being assigned a single midwife who supported them both antenatally and in some cases postnatally; additional resource and more individualised care in terms of increased numbers of visits and midwives being able and willing to make home visits or seek out women in other settings such as town centres if needed, and the highly specialised skillset of the midwives within the specialist service and their relationships with other health and social work professionals both within and outside the teams. Although some of these aspects, including continuity of care, are now a policy for mainstream antenatal care, this is still being implemented in Scotland, and faces challenges in usual care [22]. Continuity of care in midwifery-led models, involves the care of individuals by the same midwife (or team of midwives) throughout pregnancy, birth and the postnatal period [23] and has been well evidenced in mainstream populations to result in better outcomes at birth, including fewer preterm births, lower mortality before and after 24 weeks, lower use of intervention during birth, and less neonatal resuscitation [23–25]. Evidence also indicates that women feel that continuity of care provides a better experience of care during the antenatal period [26]. The high quality of antenatal care reported in these findings, in terms of having highly skilled staff, able to provide dedicated, individualised and intensive support, is key to being able to establish a therapeutic relationship between the midwife and women, which has been found to be highly valued by women [26]. This has been found to be especially important for women who use substances as fears of being reported to social services or the police, and subsequent child removal can guide service involvement for these women; the building of a trusting relationship between midwife and woman may help to allay these fears and encourage engagement in antenatal care [27, 28].

Integrated care approaches have been highlighted as critical in the care of pregnant women with substance use disorders [21]. Indeed, there appears to be a perception among health professionals interviewed that some of the models of antenatal care examined in this study do not provide enough in the way of integrated care and are thus failing to provide the appropriate support for these women, for example in relation to specific psychological services for trauma focussed therapy. The prevalence of trauma in this population is high: for example, a study of women who inject drugs found that in childhood 60.2% had been sexually abused, 55.2% physically abused, 45.9% emotionally neglected and 59.7% physically neglected [29]. Thus trauma-informed psychological support is critical for these women in supporting their, and their child’s, long-term outcomes.

Other challenges for health professionals included engaging women in antenatal services, despite the substantial efforts made by some midwives to maintain contact with women. Evidence suggests that women are more likely to miss appointments if they have recently used illicit substances [12], invoking Tudor-Hart’s [30] Inverse Care Law, whereby those perhaps most in need of support are the least likely to access or receive it. This evidence again stems from the US, however, where laws in some states are far more punitive than in the UK. However, a suggestion was made by participants that mandated drug testing was being carried out in some cases: drug testing should be done with informed consent, and this requires the purpose, interpretation and reporting of the test, including risks and benefits, to be clearly understood by the women so she can decide whether to participate [31]. If women are involved in the child protection system and are being told drug testing is mandatory, then they are being coerced and this does not meet the criteria for informed consent. Previous studies have indicated anxiety of parents around providing samples in relation to fear of losing custody of their baby when they were born [32]. It is questionable whether this level of scrutiny is beneficial for women and their infants, or for engagement with antenatal services. McGrory et al. [33] describe engagement and compliance as being at opposite ends of the spectrum, with women reporting that being compliant did not necessarily equate to being positively engaged in services.

Evidence around barriers to engaging with health services outwith antenatal services (e.g. sexual health services) for this group of women highlight other additional barriers, including structural barriers, such as lack of transportation and (in the US and similar systems) insurance, and stigma, including women’s shame and embarrassment at disclosing their substance use [34]. Stigma around substance use is often apparent through the language used. On the whole, healthcare professionals did not use stigmatising language, there was, however, mention of referral criteria being related to ‘chaotic’ substance use, and of women being referred to as ‘girls’ [35].

### Strengths and limitations

This study conducted interviews across three diverse health boards in Scotland, allowing comparison of services in different areas. Semi-structured interviews were conducted with a diverse range of health and social care staff, providing multiple viewpoints on service delivery. Conducting research in a healthcare environment still heavily affected by the Covid-19 pandemic, however, was highly challenging; the result of this was that we achieved fewer interviews in some areas than hoped, and therefore do not capture all viewpoints across all areas: notably, no drug treatment staff participated in the study. Interviews were often time limited due to work commitments, and thus people were possibly unable to go into as much depth as they would have liked. Additionally, resource meant that this study was limited to capturing data from case study areas, and health and social care workers only; other areas, particularly rural and remote areas, such as the Highlands and Islands, may demonstrate particular challenges to service implementation and delivery. Future research should include the views of women who use substances in Scotland, to provide a more rounded view of strengths and challenges of antenatal and postnatal services for women who use drugs in pregnancy.

## Conclusions

The antenatal period is particularly important for health services to engage with pregnant women in order to improve the initial and long-term health outcomes for both mother and baby. This can often be seen as a key point for women to improve their health and stabilise they substance use. This can only be done through high quality, well-resourced multi-agency provision. WHO Guidelines clearly recommend the provision of holistic and personalised care for pregnant women who use substances, including tailored psychological treatments and social supports [7]. Whilst much of this appears to be happening in the case study areas investigated in Scotland, there remains something of a postcode lottery of access to specialist services for pregnant women who use drugs, and there remains a long way to go to reach the WHO aspirations for women’s care. In Scotland, further research is needed to explore both women who use opioids’ perceptions of specialist antenatal care and barriers to accessing such care, and perceptions in rural and remote communities, which may experience particular challenges.

### Supplementary Information

**Additional file 1.** Practitioner Topic Guide; Contains questions used as basis for semi-structured interviews with professionals working with women who use opioids in pregnancy and postnatally.

## Data Availability

Data are not currently publicly available. For discussions around access to data, please contact Dr. Louise Marryat: lmarryat001@dundee.ac.uk.

## Abbreviations

MAT: Medication Assisted Treatment
NAS: Neonatal Abstinence Syndrome
NHS: National Health Service
NHS GGC: National Health Service Greater Glasgow and Clyde
SNIPS: Special Needs In Pregnancy Service
