# Supplementary material for: Antenatal care of women who use opioids: a qualitative study of practitioners’ perceptions of strengths and challenges of current service provision in Scotland
Source: BMC Pregnancy Childbirth. 2024 Jan 23;24:75. doi: 10.1186/s12884-024-06265-w (PMC10804550; doi:10.1186/s12884-024-06265-w)
Supplement: Supplementary file 1 — Additional file 1. Practitioner Topic Guide; Contains questions used as basis for semi-structured interviews with professionals working with women who use opioids in pregnancy and postnatally. [file 12884_2024_6265_MOESM1_ESM.pdf]

Additional File 1: Topic Guide

1. Would you be able to tell me a little bit about your role and how often you interact with women who use opioids during pregnancy?
   - Prompt: How long have you been in the role? Where are you based (Which Health board area)?

2. Could you tell me a little about the level of substance use in your local area?

3. What are the current antenatal pathways for women who use opioids in your area?
   - Prompt: What offer services might you refer women to during pregnancy?
   - Prompt: Do these women stay on the mainstream antenatal pathway or is there a specialist service for them?

4. From your perspective, are there any gaps in the antenatal pathway for women who use opioids during pregnancy in your area?

5. What works well in your service for women who use opioids in pregnancy?

6. In your line of work, how do you currently collect data around substance use?
   - Prompt: Is there data specifically collected about women who use opioids?
   - Prompt: What questions do you routinely ask?
   - Prompt: Will you ask additional questions on top of the routine questions?
   - Prompt: To what extent do these questions get recorded?

7. Would you be able to briefly explain how the data is currently entered?
    - Prompt: Where is it entered to?
    - Prompt: By whom?

8. What do you see as the biggest challenge/ barriers to collecting data about this group?

9. In your opinion, what are the current gaps in the data?
    - Prompt: What data needs to be collected?

10. Is there anything else you would like to touch upon, that we have not covered?
